# 420,000 year assessment of fault leakage rates shows geological carbon storage is secure

**DOI:** 10.1038/s41598-018-36974-0

**Published:** 2019-01-25

**Authors:** Johannes M. Miocic, Stuart M. V. Gilfillan, Norbert Frank, Andrea Schroeder-Ritzrau, Neil M. Burnside, R. Stuart Haszeldine

**Affiliations:** 1 School of GeoSciences, University of Edinburgh, James Hutton Road, Edinburgh, EH9 3FE UK; 2 Institute of Earth and Environmental Sciences, University of Freiburg, Albertstr. 23b, 79104 Freiburg, Germany; 3 Institute for Environmental Physics, University of Heidelberg, Im Neuenheimer Feld 229, 69120 Heidelberg, Germany; 4 School of Engineering, University of Glasgow, James Watt South Building, Glasgow, G12 8QQ UK

**Keywords:** Climate-change mitigation, Environmental impact, Geology, Tectonics

## Abstract

Carbon capture and storage (CCS) technology is routinely cited as a cost effective tool for climate change mitigation. CCS can directly reduce industrial CO_2_ emissions and is essential for the retention of CO_2_ extracted from the atmosphere. To be effective as a climate change mitigation tool, CO_2_ must be securely retained for 10,000 years (10 ka) with a leakage rate of below 0.01% per year of the total amount of CO_2_ injected. Migration of CO_2_ back to the atmosphere via leakage through geological faults is a potential high impact risk to CO_2_ storage integrity. Here, we calculate for the first time natural leakage rates from a 420 ka paleo-record of CO_2_ leakage above a naturally occurring, faulted, CO_2_ reservoir in Arizona, USA. Surface travertine (CaCO_3_) deposits provide evidence of vertical CO_2_ leakage linked to known faults. U-Th dating of travertine deposits shows leakage varies along a single fault and that individual seeps have lifespans of up to 200 ka. Whilst the total volumes of CO_2_ required to form the travertine deposits are high, time-averaged leakage equates to a linear rate of less than 0.01%/yr. Hence, even this natural geological storage site, which would be deemed to be of too high risk to be selected for engineered geologic storage, is adequate to store CO_2_ for climate mitigation purposes.

## Introduction

The integrity of engineered subsurface geological carbon dioxide (CO_2_) reservoirs is governed by a range of geological, geochemical, and geotechnical factors. Faults and naturally occurring or induced fracture networks can form preferential fluid pathways that bypass impermeable caprock seals and may lead to rapid migration of CO_2_ from the subsurface reservoir to shallow aquifers or the atmosphere^1–4^. Fracture permeability, which controls the flow rate and thus leakage rates, is dependent on the fracture aperture, the orientation of the local stress field, the dissolution and precipitation of minerals, host rock strength and permeability, the fluid type, fluid pressures and fluid flow rates^5–8^. Understanding flow of CO_2_-rich fluids through the subsurface is crucial as any leakage to the atmosphere must be below a range of 0.001–0.01% per year of the CO_2_ injected to ensure the effectiveness of carbon capture and storage as mitigation tool to avert severe climate change^9,10^.

CO_2_ derived from natural earth processes accumulates in subsurface rock formations where it can remain naturally trapped over geological time periods. These natural CO_2_ stores can be found in many sedimentary basins worldwide and provide a unique opportunity to study the environmental^11,12^ and human health^13^ impacts of leakage, as well as the long-term behaviour of, and the retention mechanisms for, CO_2_ in the subsurface^14,15^. The Colorado Plateau, south-western USA, and surrounding areas contain at least 16 predominantly magmatically sourced CO_2_ reservoirs^16,17^. Based on estimates of the timing of CO_2_ injection, these reservoirs have securely retained CO_2_ for 10^5^–10^6^ years^16–19^, comparative to the required retention timescale of CO_2_ storage in engineered sites of 10 ka^20^.

However, travertine deposits indicate CO_2_ leakage to the surface in recent geological times at several locations in the region. This includes the well-studied Green River/Crystal Geyser area where CO_2_-rich fluids are leaking to the surface through fractures in the damage zones of two normal faults, through an abandoned petroleum exploration well, and in natural, off fault springs and seeps^12,21–24^. Travertine deposits linked to tectonic structures can also provide a record of fault movement as they can be dated using U-series methods^25^. Such dates have successfully been used to reconstruct leakage histories along faults^11^, and to gain insights into the volumes of CO_2_ leaked^26^. Travertines have also been used to reveal continental-scale links between the mantle and groundwater systems^27^.

**Figure 1:**
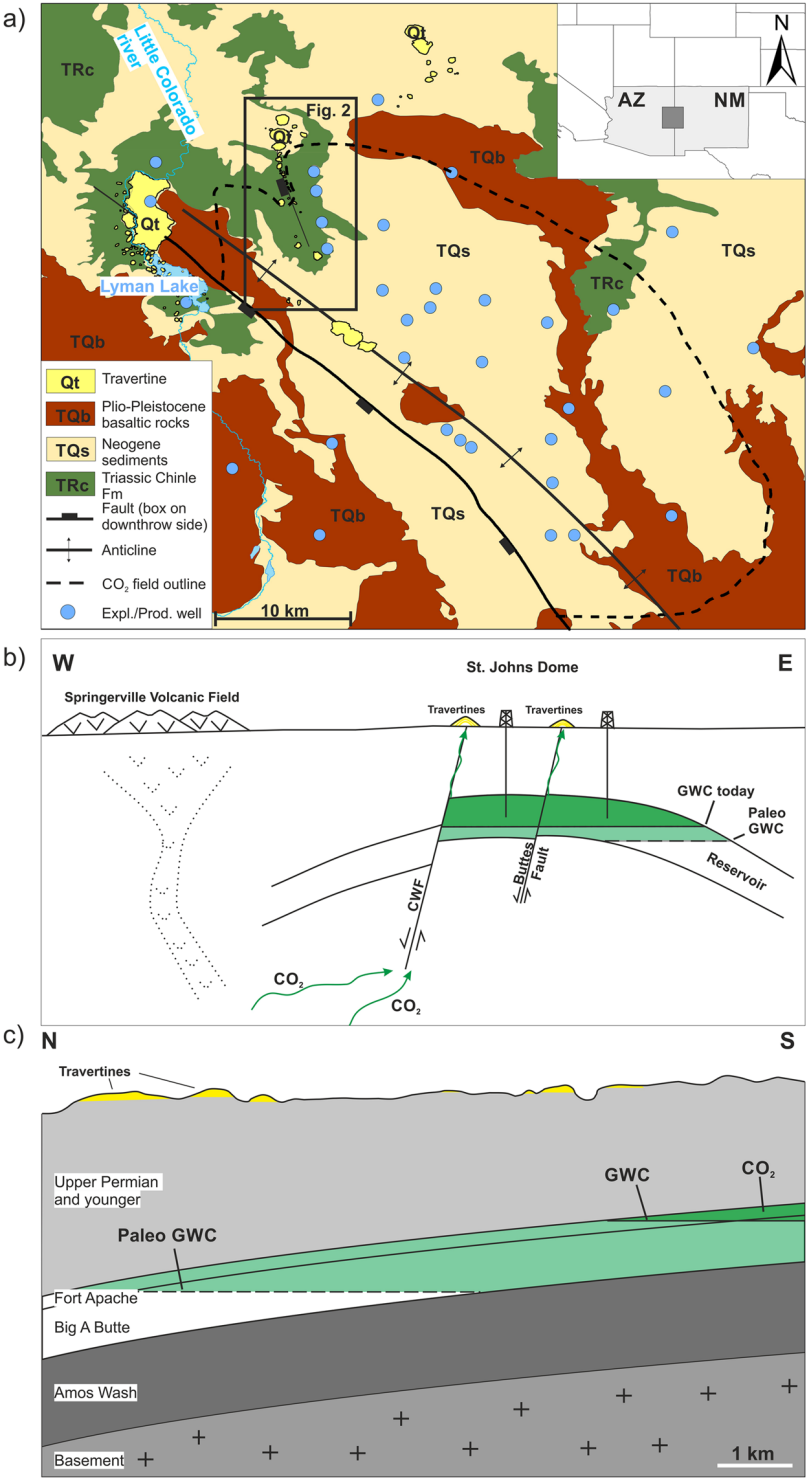
Location and geological setting of the St Johns Dome natural CO_2_ reservoir. (**a**) Map of the St. Johns Dome area showing the extent of the CO_2_ reservoir in the subsurface, orientation of the studied faults, the location of travertine deposits, and the location of exploration and production wells used to build the subsurface model. Black rectangle indicates the location of the investigated travertine deposits along the Buttes Fault, which straddles the gas water contact, see Fig. 2. Inset shows the location of the study area at the Arizona (AZ) – New Mexico (NM) border. (**b**) Sketch of the subsurface structural setting of the St. Johns Dome CO_2_ reservoir. The reservoir is located in the crest of a faulted anticline, the CO_2_ charge is associated with the nearby Springerville volcanic field. The magmatic CO_2_ migrates into the reservoir along the deep seated Coyote Wash Fault (CWF) and from there along faults to the surface where travertine precipitates. The gas-water-contact indicates the boundary between the gas-filled and the water-filled reservoir interval. The continuous CO_2_ leakage caused a shrinkage of the reservoir volume and an up-dip migration of the gas-water contact, with a paleo-GWC illustrating the original reservoir extent. (**c**) Sketch parallel to the Buttes normal fault illustrating the geological subsurface setting and the travertine deposits on the surface. Vertical exaggeration ~15x. The present day GWC is located at the southern end of the fault, towards the top of the anticline. Travertine deposits on the surface demonstrate that vertical leakage of CO_2_ occurred at the northern end of the fault in the past, highlighting that a plaeo-GWC must have been located deeper than the present day GWC.

To study how a CO_2_ storage site may leak over many thousands of years, here we examine a very large (>4.7 × 10^10^ m^3^ of recoverable CO_2_) natural accumulation, within the St Johns Dome located on the border of northern Arizona and New Mexico. At this site faults extend upwards from the reservoir, through the caprock, to the surface^27–29^. These faults are directly linked to locations of modern CO_2_ seeps^30,31^ and geologically older accumulations of travertine from past CO_2_ leakage. Here, we examine the history of CO_2_ leakage along structural elements in the region above the natural CO_2_ reservoir. The subsurface reservoir of over ~600 km^2^ fed 102 travertine mounds, which cover a surface area of more than 30 km^2^ (Fig. 1). These attest to vertical migration of CO_2_-charged fluids from the reservoir to the surface. The formally commercially exploited reservoir (>90% CO_2_)^16^ lies within rocks of the Permian Supai Formation (siltstones, sandstones and limestones) which are located in 400–700 m depth within a broad, north-west-trending asymmetrical anticline that is dissected by the steeply dipping NW-SE trending Coyote Wash fault (Fig. 1)^29^. The gas phase CO_2_ is of magmatic origin^16,30^ and the CO_2_ contained in the reservoir is associated with the nearby Springerville volcanic field where volcanic activity occurred between 8.97 ± 0.19 Ma and 0.31 ± 0.01 Ma via more than 400 separate volcanic vents^32^.

**Figure 2:**
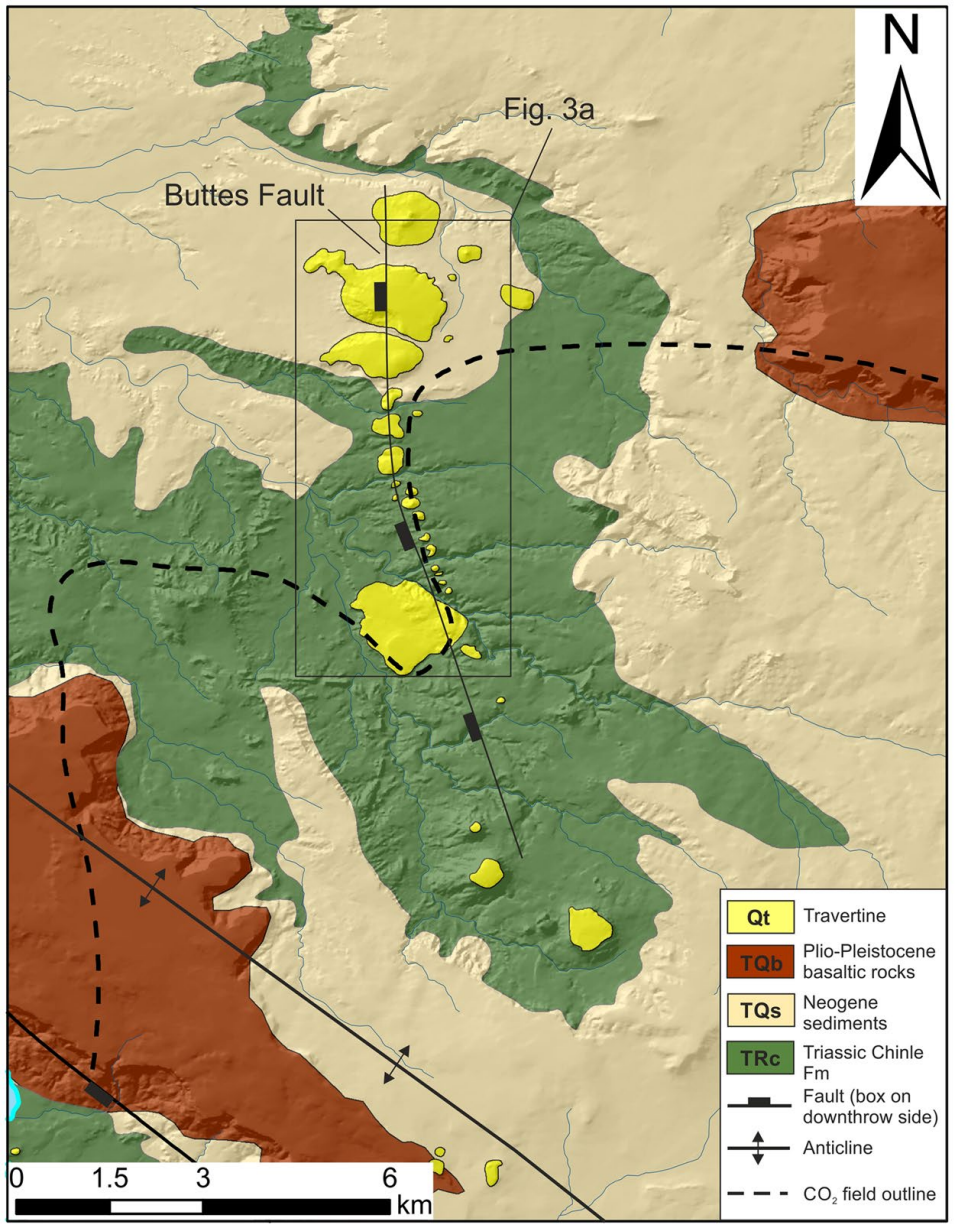
Geological map of the study area along the Buttes Fault. Note that the travertine mounds align along the fault trace. For location within the St. Johns Dome CO_2_ field see Fig. 1a.

Noble gas and water geochemistry data indicate that CO_2_ rich fluids still migrate today from the subsurface reservoir along faults to the surface^30,33^. Present day travertine formation occurs along the northern edge of a massive, 6.5 km by 3.5 km, travertine platform north of Lyman Lake (Fig. 1)^27^. Previous U-Th dating of the travertine deposits around Lyman Lake and Salado Springs reveal a history of CO_2_ leakage over the past ~350 ka^27,34^. Here, we provide new insights into the leakage history along the Buttes normal fault, a laramidic compressional fault^34^ which may be a northern extension of the Coyote Wash fault^28^, using U-Th dating of travertine mounds that align with the fault trace over a distance of more than 7 km (Figs 1 and 2). Note that the fault is poorly exposed and thus the mapped surface fault trace is associated with some spatial uncertainty (~100 m). Linking travertine ages with travertine mass enables us to estimate volumes and rates of CO_2_ leaked to the surface and, for the first time, compare those to volumes of CO_2_ stored within the reservoir.

## Travertine U-Th dates Provide a 500 ka history of CO_2_ leakage

Using samples collected from manual surface investigations, we obtained a total of 16 ages from 10 travertine mounds along the Buttes Fault using the U-Th method^35,36^, with an additional six samples exhibiting ages outside the limits (~550 ka) of the dating technique (see Methods and Supplementary Information). For the mounds where a single age was obtained the samples were taken from the stratigraphically highest point. For mounds with several ages, samples were collected in vertical cross-sections through the mounds. The intimately grown spar calcite crystals of surface-deposited carbonate samples are internally clean and pure. One sample showed limited evidence of windblown contamination (^230^Th/^232^Th activity ratios of less than 25) and was discarded. The absence of evidence of windblown contamination from local detritus and younger/older calcite material provides confidence in the quality of the samples and that reliable ages could be retrieved.

**Figure 3:**
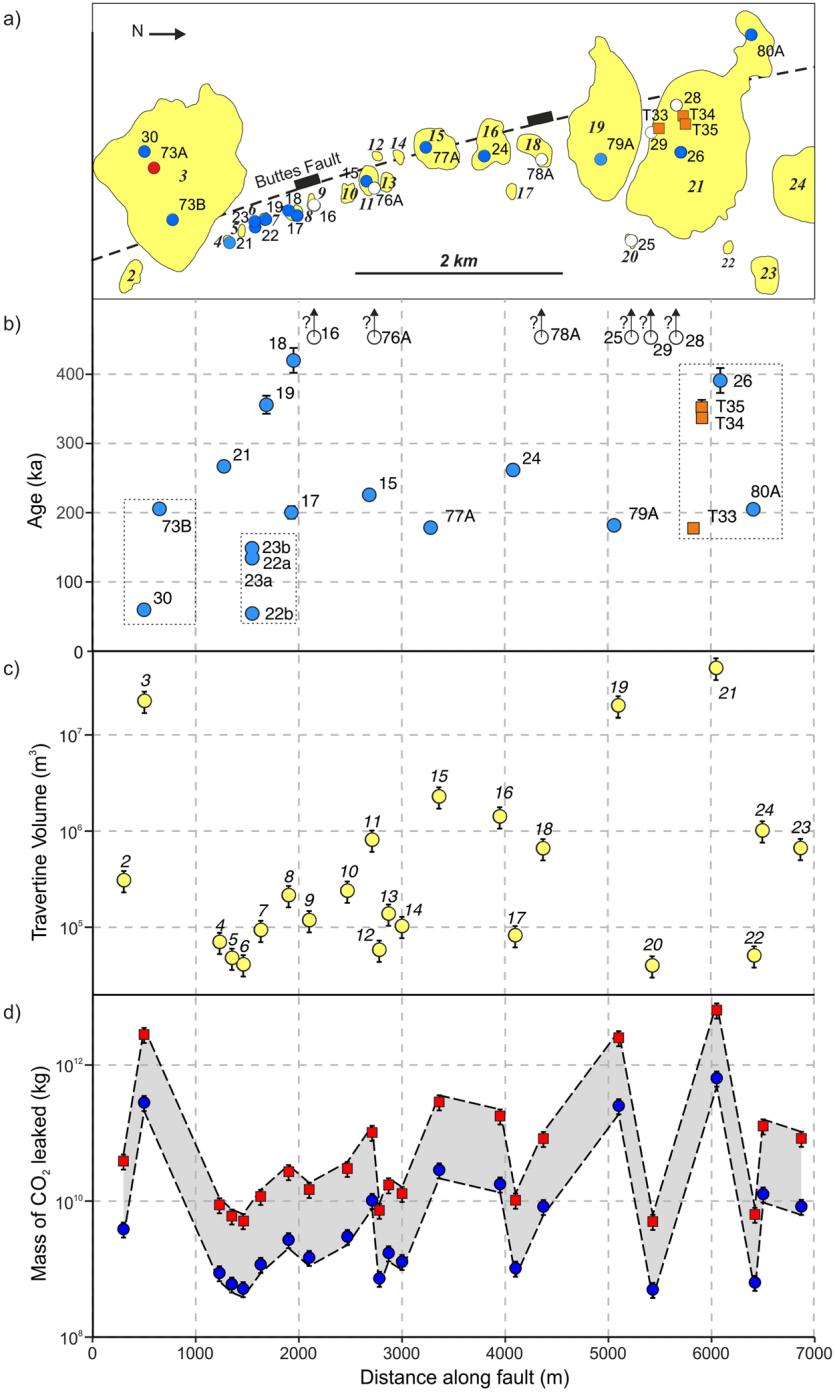
Locations, ages, and volumes of travertine mounds and mass of leaked CO_2_ along the Buttes Fault, St. Johns Dome. (**a**) Study area location map of travertine mounds based on DEM analysis and aerial photogrammetry, sample locations, and trace of the Buttes Fault. Blue circles = Samples with ages obtained in this study; white circles = outside dating limits; orange squares = data from Embid (2009)^34^. (**b**) U-Th ages of the travertine samples used in this study, error bars are smaller than the points used unless indicated, see Methods for details. Grey dotted boxes indicate samples from the same travertine mound. (**c**) Calculated volumes of the individual travertine mounds, details of the calculation method are provided in Methods. (**d**) Mass of CO_2_ leaked based on travertine volumes; red squares have been calculated assuming 1% of leaked CO_2_ is trapped in the precipitated travertines, blue circles assuming a precipitation ratio of 10%, see Methods for details.

Travertine U-Th ages range from 54 ± 1 ka to 420 ± 18 ka along the Buttes Fault, with young ages (<170 ka) only occurring at the southern part of the studied fault section (Fig. 3). U-Th ages of the northernmost studied mound (No. 21, Fig. 3) range from 391 ± 18 ka to 178 ± 2 ka, while mounds at the southern end of the fault (Nos. 2 to 8, Fig. 3) indicate that CO_2_ leakage occurred from 420 ± 18 ka until 54 ± 1 ka. Single U-Th dates cannot constrain the length of the active depositional period of each mound, nor when it was initiated or terminated. However, for the mounds with five (No. 21), four (No. 6), and two (No. 3) dated samples, the minimum lifespans can be constrained to 213 ka, 94 ka, and 145 ka, respectively. Whilst periods of inactivity cannot be ruled out, it demonstrates that fluid migration pathways along faults can stay open over several 100 ka. It is notable that within the last 150 ka travertines have only formed in the southern part of the fault and that samples exceeding the dating limits of the U-Th method are located dominantly along the northern part of the fault.

The southwards migration of CO_2_ leakage along the fault through time could be caused by a range of mechanisms, namely; (i) closure of pathways due to mineral precipitation, (ii) changes in hydrology due to climatic changes, (iii) local or triggered seismic activity on the fault, or (iv) a change in CO_2_ supply. Based on petrographic relationships of authigenic minerals and geochemical modelling of the mineral phases it has been shown that reservoir pressures were higher in the past (Moore *et al*.^29^), indicating a thicker, spatially more extensive, gas-filled reservoir interval. We suggest that the travertine mounds located outside the present day subsurface extent of the reservoir attest to this paleo-reservoir extent and that continuous leakage of large volumes of CO_2_ from the subsurface reservoir led to a depletion of the reservoir. This depletion led to a movement of the gas-water-contact (GWC) upwards to the higher parts of the anticline, cutting off the CO_2_ supply to the northern end of the Buttes Fault and thus ending travertine precipitation (Figs 1 and 2). Hence, travertine volumes combined with U-Th ages can enable the calculation of leakage volumes from the reservoir through time.

## CO_2_ leakage volume and rates

Travertine formation occurs when CO_2_-charged fluids outgas CO_2_ as they migrate to shallower depths and lower pressure, driving CaCO_3_ supersaturation and carbonate precipitation:

1 $$2{{\rm{HCO}}}_{3}^{-}({\rm{aqueous}})+{{\rm{Ca}}}^{2+}\leftrightarrow {{\rm{CaCO}}}_{3}({\rm{solid}})+{{\rm{CO}}}_{2}({\rm{gaseous}})+{{\rm{H}}}_{2}{\rm{O}}$$

**Figure 4:**
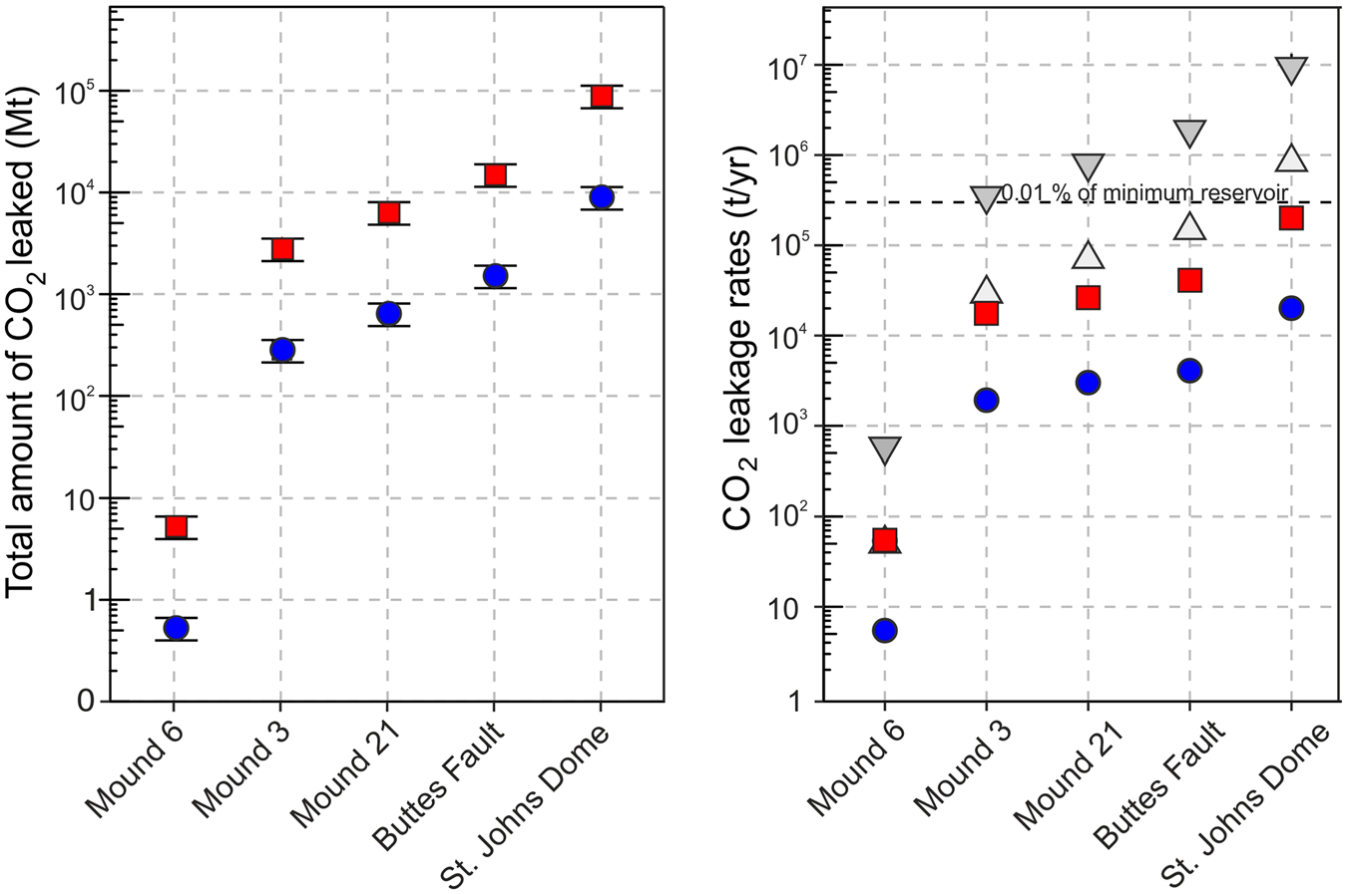
Mass of CO_2_ leaked and CO_2_ leakage rates. Left panel: Mass of CO_2_ leaked based on precipitation ratios of 10% (blue circles) and 1% (red squares) for the three mounds with constrained minimum lifespans, the Buttes Fault and the whole of St. Johns Dome area, respectively. Error bars indicate the uncertainty on estimated volumes due to the accuracy of volumetric measurements. Right panel: CO_2_ leakage rates of the same mounds and areas with time averaged leakage rates for precipitation ratios of 10% (blue circles) and 1% (red squares) as well as leakage rates over a time span of 10 ka, for precipitation rates of 10% (grey triangles) and 1% (grey inverted triangles). The dashed line indicates 0.01% of the minimum reservoir volume, and it should be noted that the majority of calculated leakage rates are less than this value. Errors are smaller than points.

Work on travertine deposits on the Colorado Plateau indicates that only between 1% and 10% of the dissolved CO_2_ is precipitated as travertine with the remainder being either vented as free gas into the atmosphere or retained in solution^1,11^. Calculating the mass of CO_2_ stored within the travertine mounds thus allows for an estimation of CO_2_ leaked to the atmosphere. We calculate the volume of each travertine mound along the Buttes Fault using mound thickness and area (Fig. 3, see details in Methods). Combining this with calcium carbonate density (2.8 g/cm^3^), the ratio of the molar mass of CO_2_ and carbonate (0.449), and the precipitation ratios, we are able to calculate the mass of CO_2_ leaked along the Buttes Fault (Figs 3 and 4). We find that CO_2_ leakage recorded at individual mounds ranges from less than 1 million tonnes (Mt) to more than 10^3^ Mt. These values can be considered as minimum values, as the erosion of the travertine mounds has not been accounted in these volume calculations.

The total amount of CO_2_ leaked along the Buttes Fault ranges from 1.5 × 10^3^ ± 0.17 × 10^3^ Mt for a precipitation ratio of 10% to 15 × 10^3^ ± 1.7 × 10^3^ Mt for a precipitation ratio of 1%. Using the same approach, estimated volumes of CO_2_ leaked into the atmosphere in the St. Johns area range from 9 × 10^3^ Mt to 90 × 10^3^ Mt (Fig. 4). This is a conservative estimate based on our calculations for travertine volumes, which are 20% lower than other published data on travertine volumes in the St. Johns Dome area^27^ due to the implementation of high-resolution DEMs and the disregarding of erosion (see Methods for details). The leaked volumes illustrate that large quantities of CO_2_ can migrate along fault zones to the surface and that individual pathways can induce significant loss of CO_2_ from subsurface reservoirs.

Combining the calculated volumes of leaked CO_2_ with the leakage history provided by the U-Th dates allows estimation of the CO_2_ leakage rates along the Buttes fault (Fig. 4). For the three mounds where the lifespans are constrained, the leakage rates range from less than 10 tonnes of CO_2_ per year (t/yr) to more than 10^4^ t/yr if constant leakage rates are assumed. The total leakage rate for the Buttes Fault in case of time averaged leakage is up to 3.6 × 10^4^ t/yr. Note that the choice of precipitation ratio is crucial as it introduces a difference in leakage rates of one order of magnitude. Assuming constant CO_2_ leakage over the last 420 ka, the travertine deposits above the St. Johns Dome CO_2_ reservoir indicate overall leakage rates of up to 2 × 10^5^ t/yr, which is an order of magnitude lower than annual injection rates at currently operating large-scale carbon storage sites of between 0.7 and 1.2 × 10^6^ t/yr^3^.

Future carbon storage sites may have significantly higher injection rates. As leakage may have occurred in pulses with hiatuses between times of travertine deposition – as reconstructed elsewhere on the Colorado Plateau^12^ – leakage rates for short pulses with an overall duration of 10 ka were also calculated (Fig. 4). These rates are generally one order of magnitude higher than the rates for constant flow and precipitation ratios of 1% and may reach up to 1.5 × 10^6^ t/yr for the Buttes Fault and up to 9 × 10^6^ t/yr for the St. Johns Dome. However, it seems unlikely that the massive travertine deposits of both the Buttes Fault and the St. Johns Dome have formed within the short time frame of 10 ka, which is less than 3% of the overall time-span recorded by the travertine U-Th ages. Thus, leakage of CO_2_ likely occurred at rates between the end-members outlined. It should be noted that all of these leakage rates do not take erosion into account and thus reflect minimum estimates of CO_2_ migration volumes.

## CO_2_ volume stored in the reservoir

To constrain the volume of CO_2_ stored within the St. Johns Dome reservoir over the past 420 ka, a 3D subsurface model was constructed (see Methods for details). The present day reservoir retains ~1.9 × 10^3^ Mt of CO_2_ while a paleo-reservoir, which had a larger subsurface extent and a GWC located a minimum of 50 m deeper than the present GWC (constrained by the occurrence of travertine mounds on the surface, assuming predominately vertical buoyancy driven migration of the migrating CO_2_), held ~2.7 × 10³ Mt of CO_2_. Both volumes are between one to two orders of magnitude lower than the estimated amount of CO_2_ leaked over the last 420 ka for the whole St. Johns area, indicating constant recharging of the reservoir from a deep mantle source over its history, consistent with previous work^16,30^. The reconstructed leakage rates for individual mounds along the Buttes Fault represent an annual volume loss of less than 10^−2^ to 10^−6^% of the total CO_2_ retained in the reservoir, while the leakage rates for the Buttes Fault and the St. Johns Dome represent an annual volume loss of less than 0.1 to 0.001% of the total reservoir volume. These compare well to the limits stipulated by others^20^.

Vertical migration of fluids is often related to fracture networks that are located (1) in damage zones either side of fault cores^2,23,37,38^, which are often the widest at fault steps or beds^39^, (2) at the tip of faults^40^, and (3) at the crest of anticlines^41^. Field evidence of the travertine outcrop locations indicates that CO_2_ leakage from the St. Johns Dome reservoir has previously occurred at all three types of fracture networks (tip of Coyote Wash Fault, damage zone of Buttes Fault, crest of Cedar Mesa anticline, Fig. 1). However, the considerably larger volumes of travertines located at the tip of the Coyote Wash Fault and along the Buttes Fault illustrate that the majority of CO_2_ leakage occurred at fault induced fracture networks. The long lifespan of the studied travertine mounds highlights that CO_2_ flow through fracture networks does not necessarily lead to self-sealing due to mineral precipitation as observed elsewhere^24,42^ and high rates of flow can be sustained over geological times. Factors governing fluid flow through fracture networks at the study site could be of geomechanical nature as the continuous influx of magmatic CO_2_ into the shallow reservoir, which is needed to sustain the high leakage rates, could increase pore pressure and potentially bring fractures closer to failure and increase the fracture permeability^5,43^. The paleo-reservoir described above had a thicker CO_2_ column and pore pressures that were about 1 MPa higher compared to current reservoir pressures – possibly enough to force fractures into failure.

Importantly, whilst the St. Johns Dome has experienced a 400 ka history of large volumes of CO_2_ leakage (up to 9 × 10^4^ Mt) through structural elements (faults and crest of anticlines), the average annual loss of CO_2_ from the reservoir is <0.01% of the total reservoir volume. Annual leakage rates of <0.1% are thought to make geological carbon storage an effective tool to reduce the impact of global warming (<1,000 years)^44,45^, while annual leakage rates of <0.01% would significantly increase the timescale for which the application of CCS is beneficial^10,46^. More recent studies have shown that even high leakage rates and associated economic costs will not interfere with the effectiveness of policies for climate change mitigation^47,48^. This illustrates that large-scale vertical fluid migration through fault zones as found in this study does not necessarily impact the suitability of carbon storage sites. Therefore, a careful selection of storage sites is crucial and available site selection criteria^15,49,50^ indicate, that due to the faulting connecting the subsurface reservoir with the surface and the shallow depth of the reservoir, the studied St. Johns Dome site would not be chosen for geological storage of CO_2._ Yet, even for this natural geological storage site that would be deemed to be of too high risk for engineered storage, CO_2_ leakage rates are low enough to make this a viable CO_2_ store.

While leakage along faults from CO_2_ storage sites does not necessarily impact storage suitability of a site from a climate mitigation point of view, migration of CO_2_ through the subsurface has the potential to negatively impact shallow potable aquifers^51^, fauna and flora^52^, and humans^13^ on the surface. In particular the introduction of CO_2_-rich waters into potable aquifers is an issue, which could mobilise organic contaminants based on experimental results that show that CO_2_ addition can potentially release toxic elements from the rock^53–55^. Additionally, fear of migration of CO_2_ to the surface is the main driver of negative public opinion towards CCS and has led to the delay of storage project development^56,57^. While storing CO_2_ in offshore reservoirs can address some of these issues, site monitoring and leakage detection are more difficult compared to onshore sites. Thus acceptable leakage rates along faults may be lower than the rates required for successful climate change mitigation.

The St. Johns Dome reservoir is a very large natural storage site, which continues to accumulate CO_2_ today, from the residue of recent volcanic activity. The reservoir has retained an at least 50 m thicker CO_2_ column in the past and leakage vertically along geological faults enables rapid migration of CO_2_ from the reservoir to the surface and atmosphere. Precipitation of travertine on the land surface captures 1% to 10% of leakage, and dating of these deposits with the U-Th method reveals a leakage history from 420 ka to present. Combining travertine volumes at the surface with U-Th dates suggests that time-averaged leakage rates are less than 0.01% per year, highlighting that even this faulted site can perform adequately as a CO_2_ store.

## Methods

Travertine samples were obtained from 11 discrete travertine mounds along the Buttes Fault. Samples collected from the stratigraphically highest point are assumed to represent the youngest deposit. Samples were cut in half and the interior of the sample was inspected for areas with no visible indications of detrital material or alteration, which were then selected for U-Th dating. Samples of 300 mg were cut from the suitable areas and dating was carried out at the Institute for Environmental Physics at the University of Heidelberg, Germany using the methods of Douville *et al*.^35^ and a Thermo Fisher Scientific Xseries^II^ ICP-QMS. See supplementary information for analytical parameters and settings. Overall low to moderate ^232^Th concentrations highlight the suitability of these samples for U-series dating (Supplementary Table 1). As ^230^Th/^232^Th activity ratios of 15–20 are the upper limits for indicating the presence of non-radiogenic ^230^Th, two samples with ^230^Th/^232^Th activity ratios of <25 were discarded. Ages were calculated using an iterative Monte Carlo approach based on the disequilibrium age function from Ivanovich and Harmon^58^. Four samples exhibit ages outside the dating-range of the U-Th method and two fall just within the dating range but have very large errors associated with them and have thus not been used for the calculation of leakage rates (Supplementary Fig. 1, Table 1).

Travertine mound volumes along the Buttes Fault were calculated using surface areas, based on field mapping, a 1/3-arcsecond DEM, and mound thickness, measured during fieldwork (Supplementary Fig. 2, Table 2). We assume an error of ±10% in the volume calculations due to uncertainties in the morphology of the original land surface underneath the travertine mounds, resulting in difficulties in estimating mound dimensions. For the volume calculation for all travertine deposits in the St. Johns, the surface area was estimated from our results for the Buttes Monocline and the mapping work of Embid^34^ for the remainder of the area. An average height of 25 m for all travertine deposits was assumed based on DEM analysis and field observations. The mass of leaked CO_2_ was calculated from the mass of CO_2_ currently stored in the travertine deposits, which is calculated using the density of travertine (assumed to be entirely CaCO_3_) and the molar masses of CO_2_ (44 g/mol) and CaCO_3_ (100.1 g/mol), using a precipitation ratio of 1% and 10% (Supplementary Table 2). Leakage rates are calculated based on the minimum lifespan given by the U-Th dating for each mound. Leakage rates for the Buttes Fault are based on a lifespan of 371 ka, whilst those for the whole of the St. Johns area are based on continuous CO_2_ leakage over 450 ka.

The 3D model of the St. Johns area was built in Move^TM^, using a geological map of the area and well data (well logs, horizon markers) of 37 exploration and production wells as input data (Supplementary Table 3) and a previously published reservoir horizon map (Rauzi, 1999^28^). Constant thickness of the stratigraphic layers between wells was assumed. The stratigraphic layers of the model were populated with porosity and permeability values within the ranges given by Rauzi^28^. The current gas-water-contact is at 1494 m above sea level and is assumed to be horizontal (Supplementary Fig. 3). For the CO_2_ volume calculations, a CO_2_ density of 185 kg/m^3^ (based on reservoir conditions) and a CO_2_ saturation of 80% in the gas cap (assuming 20% of the pore space occupied by connate and residual water) was used.

## Supplementary information

Supplementary Data
