# Author Correction: 420,000 year assessment of fault leakage rates shows geological carbon storage is secure

**DOI:** 10.1038/s41598-020-60512-6

**Published:** 2020-02-20

**Authors:** Johannes M. Miocic, Stuart M. V. Gilfillan, Norbert Frank, Andrea Schroeder-Ritzrau, Neil M. Burnside, R. Stuart Haszeldine

**Affiliations:** 1 School of GeoSciences, University of Edinburgh, James Hutton Road, Edinburgh, EH9 3FE UK; 2 Institute of Earth and Environmental Sciences, University of Freiburg, Albertstr. 23b, 79104 Freiburg, Germany; 3 Institute for Environmental Physics, University of Heidelberg, Im Neuenheimer Feld 229, 69120 Heidelberg, Germany; 4 School of Engineering, University of Glasgow, James Watt South Building, Glasgow, G12 8QQ UK

Correction to: *Scientific Reports* 10.1038/s41598-018-36974-0, published online 25 January 2019

This Article contains errors in the Reference list. Reference 3 is incorrectly listed as ‘Song, J. & Zhang, D. Comprehensive Review of Caprock-Sealing Mechanisms for Geologic Carbon Sequestration. **47**, 9–22 (2012)’. The correct reference is listed below as ref. 1.

Reference 16 is incorrectly listed as ‘Gilfillan, S. M. V. *et al*. The noble gas geochemistry of natural CO_2_ gas reservoirs from the Colorado Plateau and Rocky Mountain provinces. *USA*. **72**, 1174–1198 (2008)’. The correct reference is listed below as ref. 2.

Reference 20 is incorrectly listed as ‘Alcalde, J. *et al*. Quantifying geological CO_2_ storage security to deliver on climate mitigation (2018)’. The correct reference is listed below as ref. 3.

Reference 32 is incorrectly listed as ‘Condit, C. D. & Connor, C. B. Recurrence rates of volcanism in basaltic volcanic fields: An example from the Springerville volcanic field. *Arizona*. **108**, 1225–1241 (1996)’. The correct reference is listed below as ref. 4.
