# Supplementary material for: 420,000 year assessment of fault leakage rates shows geological carbon storage is secure
Source: Sci Rep. 2019 Jan 25;9:769. doi: 10.1038/s41598-018-36974-0 (PMC6347600; doi:10.1038/s41598-018-36974-0)
Supplement: Supplementary file 1 — Supplementary Data [file 41598_2018_36974_MOESM1_ESM.pdf]

Supplementary Data for

420,000 year assessment of fault leakage rates shows geological carbon storage is secure

Authors:

Johannes M. Miocic[1,2*], Stuart M.V. Gilfillan[1], Norbert Frank[3], Andrea Schroeder-Ritzrau[3], Neil M. Burnside[4], Stuart Haszeldine[1]

[1]School of GeoSciences, University of Edinburgh, James Hutton Road, Edinburgh, EH9 3FE, UK

[2]Institute of Earth and Environmental Sciences, University of Freiburg, Albertstr. 23b, 792104 Freiburg, Germany

[3]Institute for Environmental Physics, University of Heidelberg, Im Neuenheimer Feld 229, 69120 Heidelberg, Germany

[4]School of Engineering, University of Glasgow, James Watt South Building, Glasgow G12 8QQ, UK

*Corresponding author: Johannes.miocic@geologie.uni-freiburg.de

Supplementary Figures

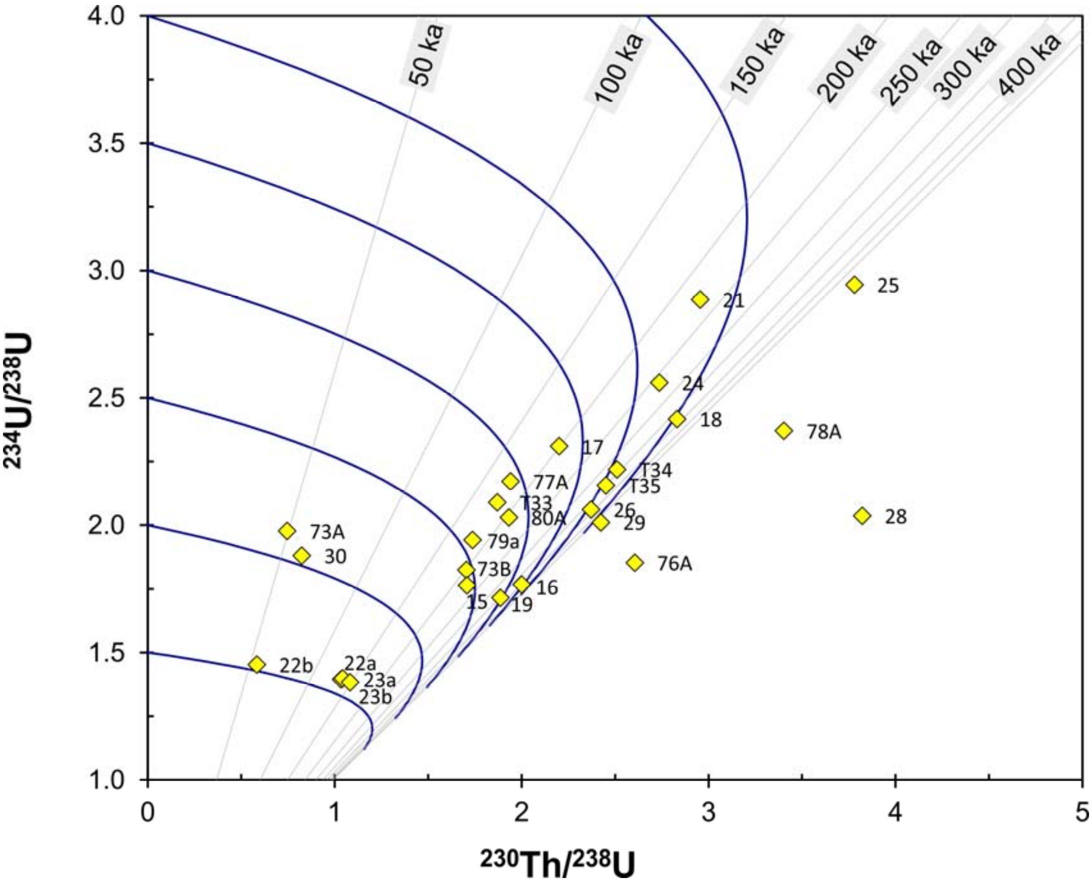

Supplementary Figure 1: $^{230}Th/^{238}U$ activity ratio vs $^{234}U/^{238}U$ activity ratio isochron plot of travertine samples of the Buttes Fault. Blue lines are evolution curves, straight grey lines are isochrones in 50 ka intervals. 2σ errors are smaller than data points. Note that four samples (25, 28, 76A, 78A) are located outside the dating-range of the method.

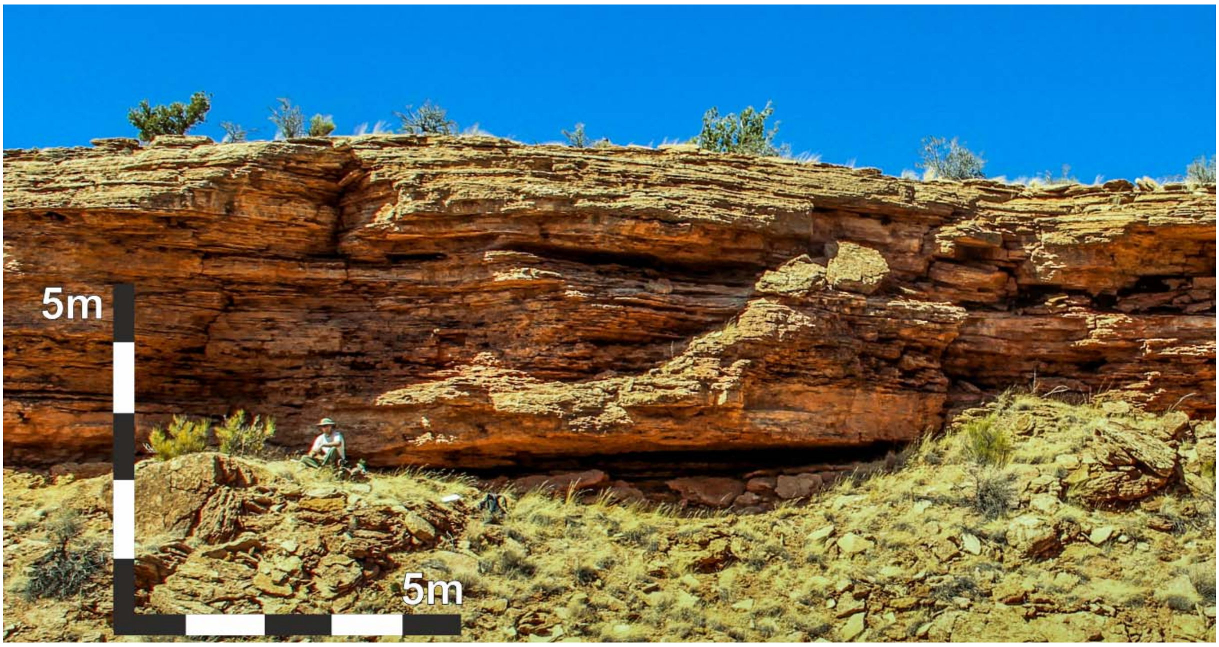

Supplementary Figure 2: Field photograph showing travertine mound No. 5. Note person for scale. Thickness of mound is 5 m.

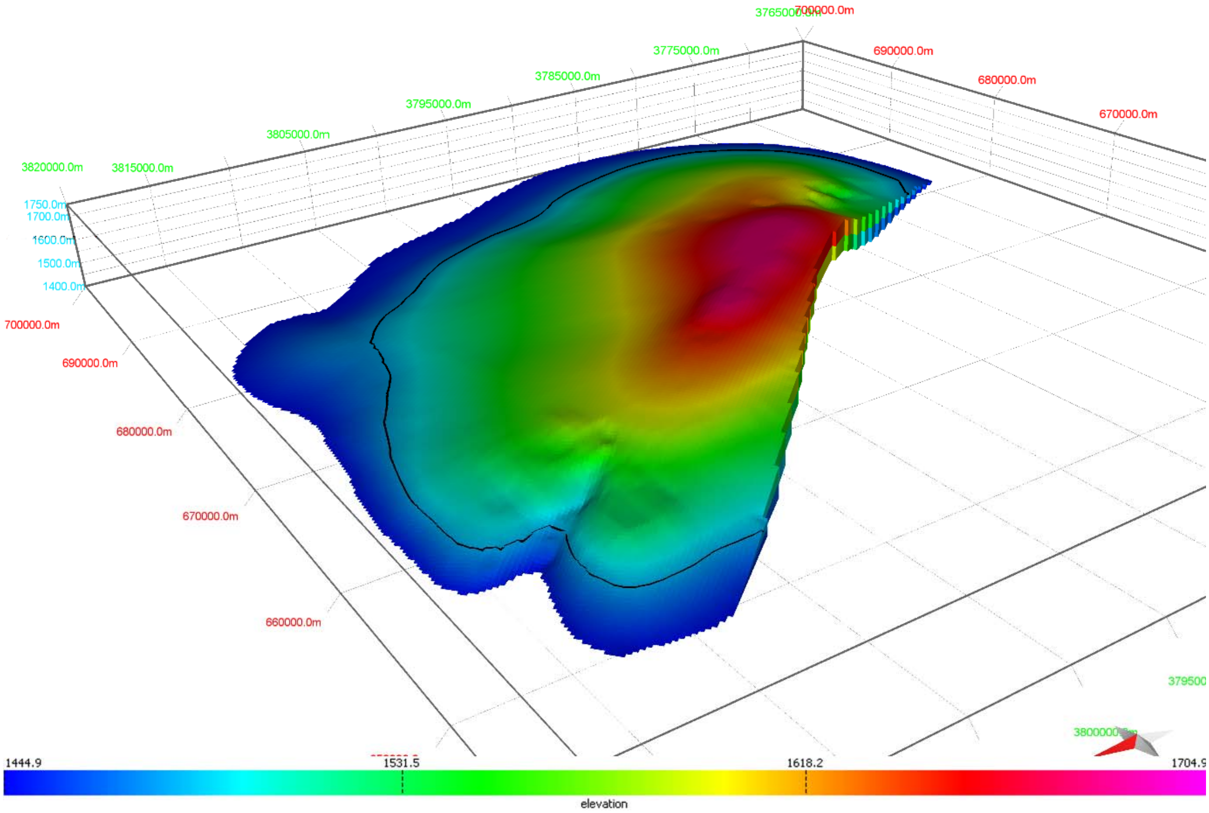

Supplementary Figure 3: Figure showing the reservoir volume of the St. Johns Dome assumed to be filled with CO_2 in the past, assuming paleo-gas water contact of 1445 m above sea level. Black line indicates present day GWC. Model is 15x vertical exaggerated.

Supplementary Tables

Supplementary Table 1: Table of U-Th dating results of travertine samples of the Butttes Fault, St. Johns Dome. Activity ratios indicated in squarded brackets, OOR = out of range, all ± = 2σ errors. # indicates samples with low 230Th/232Th activity ratio, T indicates samples from Embid (2009).

Sample	^{238}U (ppb) ± (ppb)	^{232}Th (ppb) ± (ppb)	$^{230}Th/^{238}U$ (activity)	$^{230}Th/^{232}Th$ (atomic)	$d^{234}U$ (‰) ± (‰)	Age (ka) ± (ka)	Age_{corr} (ka) ± (ka)	$d^{234}U_{ini}$ (‰) ± (‰)
15	538.2 ± 1.6	20.35 ± 0.10	1.708 ± 0.010	137.83 ± 0.95	757.0 ± 5.0	225.8 ± 3.9	225.9 ± 3.9	1433 ± 18
16	266.20 ± 0.31	8.272 ± 0.037	2.068 ± 0.015	205.3 ± 1.7	761.0 ± 5.8	ODR		
17	1225.8 ± 1.4	40.20 ± 0.73	2.200 ± 0.044	207.1 ± 5.6	1307 ± 10	200.7 ± 8.9	200.4 ± 9.0	2302 ± 60
18	1015.9 ± 1.1	107.66 ± 0.61	2.832 ± 0.018	82.45 ± 0.70	1377.5 ± 4.2	420 ± 18	420 ± 18	4509 ± 226
19	838.3 ± 1.9	2.066 ± 0.015	1.887 ± 0.013	2338 ± 22	714.9 ± 4.7	356 ± 13	356 ± 13	1955 ± 76
21	810.43 ± 0.75	261.4 ± 1.8	2.96 ± 0.020	27.87 ± 0.27	1726.6 ± 4.2	268.4 ± 5.8	267 ± 6.0	3667 ± 62
24	3714.2 ± 9.1	2.373 ± 0.020	2.736 ± 0.025	13081 ± 157	1560.5 ± 5.9	261.8 ± 7.1	261.8 ± 7.1	3271 ± 64
25	637.55 ± 0.72	241.3 ± 1.3	3.65 ± 0.020	29.37 ± 0.23	1788.2 ± 4.8	ODR		
26	577.9 ± 1.5	11.278 ± 0.094	2.371 ± 0.017	371.1 ± 3.9	1056.0 ± 6.4	391 ± 18	391 ± 18	3187 ± 154
28	392.48 ± 0.35	10.347 ± 0.052	3.822 ± 0.022	441.1 ± 3.4	1029.9 ± 6.6	ODR		
29	177.45 ± 0.58	12.089 ± 0.088	2.423 ± 0.019	109.0 ± 1.1	993.1 ± 9.0	ODR		
30	588.2 ± 1.5	24.97 ± 0.24	0.8242 ± 0.0061	59.51 ± 0.71	870.8 ± 5.7	60.31 ± 0.69	59.8 ± 0.70	1031.0 ± 7.1
22a	2475.4 ± 9.6	23.13 ± 0.10	1.0424 ± 0.0060	343.7 ± 2.1	398.4 ± 6.7	135.4 ± 2.0	135.3 ± 2.0	584 ± 10
22b	3151.8 ± 7.2	7.824 ± 0.035	0.5833 ± 0.0032	723.9 ± 4.9	452.3 ± 3.1	54.44 ± 0.37	54.40 ± 0.39	527.5 ± 3.6
23a	2239.9 ± 5.3	169.74 ± 0.67	1.0336 ± 0.0048	42.02 ± 0.24	386.9 ± 3.2	135.6 ± 1.4	134.3 ± 1.6	565.5 ± 5.4
23b	2293.0 ± 4.1	3.780 ± 0.016	1.0817 ± 0.0063	2012 ± 14	382.8 ± 3.6	148.7 ± 1.9	148.7 ± 1.9	582.8 ± 6.3
#73A	814.9 ± 1.1	135.91 ± 0.55	0.7465 ± 0.0046	13.71 ± 0.10	934.5 ± 3.9	51.03 ± 0.42	48.9 ± 1.2	1072.8 ± 5.7
73B	524.86 ± 0.68	29.52 ± 0.20	1.705 ± 0.011	92.89 ± 0.84	812.2 ± 5.3	206.2 ± 3.1	205.6 ± 3.8	1452 ± 18
76A	684.42 ± 0.72	90.34 ± 0.56	2.605 ± 0.020	60.49 ± 0.59	823.4 ± 5.3	ODR		
77A	789.9 ± 1.0	35.54 ± 0.22	1.941 ± 0.013	132.2 ± 1.2	1158.3 ± 5.4	178.9 ± 2.5	178.5 ± 2.9	1918 ± 18
78A	247.26 ± 0.35	17.302 ± 0.076	3.483 ± 0.024	151.5 ± 1.2	1345.4 ± 7.2	ODR		
79A	804.80 ± 0.80	21.34 ± 0.12	1.737 ± 0.011	199.4 ± 1.7	935.6 ± 4.5	182.1 ± 2.7	181.8 ± 2.7	1564 ± 14
80A	487.76 ± 0.56	13.53 ± 0.07	1.932 ± 0.018	211.9 ± 2.2	1030.7 ± 4.8	205.3 ± 4.6	205.0 ± 4.6	1840 ± 24
T33	440.5 ± 1.1	47.47 ± 0.14	1.87 ± 0.009	53.15 ± 0.26	1089.4 ± 1.3		177.8 ± 1.9	1800.5 ± 9.6

T34	598.6 ± 1.5	12.701 ± 0.052	2.51 ± 0.01	361.3 ± 1.8	1219.1 ± 1.5		336.6 ± 5.8	3156 ± 53
T35	420.9 ± 1.0	12.368 ± 0.074	2.45 ± 0.016	255.2 ± 2.2	1156.8 ± 1.9		352 ± 11	3130 ± 100

Supplementary Table 2: Table of travertine volume, CO_2 mass, and mass of CO_2 leaked to the atmosphere for the studied mounds of the Buttes Fault and the whole St. Johns Dome area.

No.	Area (m^2)	Height (m)	Volume (m^3)	±error	Mass CO_2 (kg)	±error	Leaked CO_2 PR10% (kg)	±error	Leaked CO_2 PR1% (kg)	±error
2	40,346	7.6	3.1E+05	3.1E+04	3.9E+08	3.9E+07	3.9E+09	3.9E+08	3.9E+10	3.9E+09
3	1,465,047	15.2	2.2E+07	2.2E+06	2.8E+10	2.8E+09	2.8E+11	2.8E+10	2.8E+12	2.8E+11
4	7,667	9.1	7.0E+04	7.0E+03	8.8E+07	8.8E+06	8.8E+08	8.8E+07	8.8E+09	8.8E+08
5	6,282	7.6	4.8E+04	4.8E+03	6.0E+07	6.0E+06	6.0E+08	6.0E+07	6.0E+09	6.0E+08
6	4,479	9.1	4.1E+04	4.1E+03	5.1E+07	5.1E+06	5.1E+08	5.1E+07	5.1E+09	5.1E+08
7	8,755	10.7	9.3E+04	9.3E+03	1.2E+08	1.2E+07	1.2E+09	1.2E+08	1.2E+10	1.2E+09
8	17,616	12.2	2.1E+05	2.1E+04	2.7E+08	2.7E+07	2.7E+09	2.7E+08	2.7E+10	2.7E+09
9	12,903	9.1	1.2E+05	1.2E+04	1.5E+08	1.5E+07	1.5E+09	1.5E+08	1.5E+10	1.5E+09
10	19,607	12.2	2.4E+05	2.4E+04	3.0E+08	3.0E+07	3.0E+09	3.0E+08	3.0E+10	3.0E+09
11	37,973	21.3	8.1E+05	8.1E+04	1.0E+09	1.0E+08	1.0E+10	1.0E+09	1.0E+11	1.0E+10
12	6,350	9.1	5.8E+04	5.8E+03	7.3E+07	7.3E+06	7.3E+08	7.3E+07	7.3E+09	7.3E+08
13	18,106	7.6	1.4E+05	1.4E+04	1.7E+08	1.7E+07	1.7E+09	1.7E+08	1.7E+10	1.7E+09
14	9,614	10.7	1.0E+05	1.0E+04	1.3E+08	1.3E+07	1.3E+09	1.3E+08	1.3E+10	1.3E+09
15	124,788	18.3	2.3E+06	2.3E+05	2.9E+09	2.9E+08	2.9E+10	2.9E+09	2.9E+11	2.9E+10
16	116,051	12.2	1.4E+06	1.4E+05	1.8E+09	1.8E+08	1.8E+10	1.8E+09	1.8E+11	1.8E+10
17	10,794	7.6	8.2E+04	8.2E+03	1.0E+08	1.0E+07	1.0E+09	1.0E+08	1.0E+10	1.0E+09
18	61,880	10.7	6.6E+05	6.6E+04	8.3E+08	8.3E+07	8.3E+09	8.3E+08	8.3E+10	8.3E+09
19	657,304	30.5	2.0E+07	2.0E+06	2.5E+10	2.5E+09	2.5E+11	2.5E+10	2.5E+12	2.5E+11
20	8,721	4.6	4.0E+04	4.0E+03	5.0E+07	5.0E+06	5.0E+08	5.0E+07	5.0E+09	5.0E+08
21	1,398,573	36.6	5.1E+07	5.1E+06	6.4E+10	6.4E+09	6.4E+11	6.4E+10	6.4E+12	6.4E+11
22	8,307	6.1	5.1E+04	5.1E+03	6.4E+07	6.4E+06	6.4E+08	6.4E+07	6.4E+09	6.4E+08
23	132,696	7.6	1.0E+06	1.0E+05	1.3E+09	1.3E+08	1.3E+10	1.3E+09	1.3E+11	1.3E+10

24	617,994	30.5	1.9E+07	1.9E+06	2.4E+10	2.4E+09	2.4E+11	2.4E+10	2.4E+12	2.4E+11
25	72,557	9.1	6.6E+05	6.6E+04	8.3E+08	8.3E+07	8.3E+09	8.3E+08	8.3E+10	8.3E+09
STJ	28,832,321	25	7.2E+08	7.2E+07	9.1E+11	9.1E+10	9.1E+12	9.1E+11	9.1E+13	9.1E+12

Supplementary Table 3: Table of wells used to build the 3D model. Depths in meters below surface. Fm. = formation, Mb.= member, Ls= limestone, ss=sandstone.

Well_name	Easting	Northing	Elevation (m)	Chinle Fm.	Moenkopi Fm.	San Andres Ls	Glorieta Ss	Corduroy Mb.	Ft. Apache Mb.	Big A Butte Mb.	Raven marker	Amos Wash Mb.	Riggs Zone	Precambrian	TD
1 Federal State	650906	3800980	1848.3	-	3.4	45.7	155.4	220.1	445.6	474.6	-	-	-	-	514.2
1 Merrill	670849	3789210	2143.7	-	3.4	-	-	229.8	440.1	468.8	-	-	-	-	480.7
1 Plateau Cattle	659540	3812070	1943.7	1.5	60.4	64.9	146.3	209.7	416.4	437.7	489.5	520.6	609.6	652.3	741.0
10-02-30X	671450	3796344	2162.9	-	-	-	-	-	-	-	-	-	-	-	573.0
10-16-31	677536	3792590	2212.8	111.3	-	251.8	322.5	385.9	567.5	595.0	646.8	679.1	775.7	802.2	831.5
10-22	669129	3790637	2131.2	4.3	-	52.4	146.3	217.0	431.3	460.9	509.0	541.9	635.2	676.7	722.0
10-26-29	661188	3789369	2117.1	132.0	-	268.2	358.4	426.7	649.2	677.3	-	765.0	866.9	961.9	975.4
10-29-31	676373	3789992	2228.1	-	-	-	-	-	565.4	591.6	673.6	-	-	-	874.5
10-5-30	666069	3796611	2127.8	-	-	-	-	-	474.9	503.5	581.6	-	-	-	792.5
11-16-30	668153	3802445	2099.8	-	-	-	-	-	-	474.3	-	-	-	-	724.5
11-18	664468	3802237	2085.4	-	-	-	-	-	-	-	-	-	-	-	772.7
11-21	667783	3800849	2112.9	71.6	-	136.2	203.6	271.3	488.6	515.1	570.6	602.3	701.0	731.5	734.6
11-23-30	671562	3801527	2127.2	-	-	-	-	-	509.0	534.0	589.2	621.2	-	-	777.2
11-29-30	666198	3799763	2109.2	-	-	-	-	-	509.0	536.4	-	-	-	-	580.9
11-29-31	675793	3799706	2144.0	-	-	-	-	-	557.8	582.8	637.6	665.7	-	-	868.7
11-29-31X	675918	3799734	2143.7	-	-	-	-	-	558.4	-	-	-	-	-	612.6
1-16	690234	3797889	2105.3	-	-	-	371.2	432.8	648.6	669.3	727.9	762.0	847.3	885.7	901.6
12-15-30	669063	3812149	2050.1	104.9	-	177.4	259.1	329.8	536.4	556.0	613.3	643.7	734.0	760.5	793.4
12-16-28	648605	3812676	1829.4	4.3	-	46.3	140.2	198.1	399.3	426.1	492.6	541.0	-	-	694.9

12-31-30	664442	3807678	2032.1	-	-	72.8	152.4	-	-	-	523.6	-	-	-	707.4
12-34-29X	660165	3807848	1998.0	-	-	-	-	-	400.8	424.6	481.0	507.5	-	-	929.0
13-36-29	663858	3818142	1884.0	27.1	-	87.8	170.1	235.3	457.8	481.6	556.3	599.2	679.1	737.6	748.0
1-4	680463	3800909	2090.9	-	-	148.7	239.6	303.6	534.0	555.7	613.3	651.1	745.5	774.2	774.2
2-13 St	684425	3807486	1988.5	-	-	157.9	237.7	306.3	529.7	548.6	605.9	640.1	740.1	779.7	828.1
22-1x	659725	3810442	1952.9	3.4	-	45.1	132.6	195.1	393.2	417.0	471.8	503.5	593.8	627.9	655.0
3-1	660573	3805626	2051.0	0.9	-	71.9	165.5	227.1	433.4	462.1	513.0	545.0	2051.0	2051.0	552.6
36-1	684842	3783744	2304.3	-	-	362.1	437.4	497.4	694.9	722.4	771.1	803.5	902.2	940.3	966.2
36-2	684194	3794145	2231.7	-	-	-	403.3	466.3	673.0	697.4	753.8	791.9	890.0	929.0	978.7
9-21	678124	3782005	2198.2	132.3	-	217.0	282.9	346.9	552.3	579.1	628.5	659.0	755.3	807.7	821.1
9-22-29	660455	3781706	2192.1	150.0	-	298.7	388.3	457.2	671.8	704.7	755.3	785.8	894.3	969.3	969.3
9-5-31	676515	3786346	2250.0	-	-	-	-	-	591.9	618.7	-	-	-	-	905.0
A-1 State	654303	3787160	2047.0	-	-	181.4	292.6	-	-	-	-	-	-	-	326.7
CDV #1 State	648279	3809378	1858.1	-	1.8	-	-	211.8	412.1	438.9	509.6	554.7	-	786.4	1004.9
MaeB #1 State	676506	3781937	2217.1	-	-	236.2	326.1	409.3	610.2	636.4	2217.1	726.9	869.3	2217.1	890.3
Skelly 1 Goes	680788	3814075	1943.7	-	59.4	129.5	206.3	0.0	471.5	-	-	-	-	781.8	793.1
Skelly 1 M.N.	695735	3809187	2040.9	85.3	-	272.5	352.0	442.6	637.6	657.8	-	-	-	-	720.9

Supplementary Table 4: Analytical setting and parameters of the ICP-QMS Xseries.

Parameters	ICP-QMS Xseries
Gas flow	Cool gas: 13 l/min<br>Auxiliary gas: ~0.83 l/min<br>Sample gas: ~0.90 l/min
Sensitivity (^{238}U)	Standard: > 300.000 cps/ppb
Backgrounds with 0.5 N HNO_3	^{228}Bkg; ^{230}Th; ^{234}U: ≤0.5 cps
Oxides (Ce) and double charge ions (Ba)	<3%
Spray chamber	Corps Impact Bead Peltier
Spray and flow rate	Self-aspirating PFA nebuliser: 100 µl/min
Cones	Ni sample and skimmer cones
Detector	Electron multiplier ETP (discrete dynodes)
Signals	Gaussian picks
Standard resolution	0.7 amu
Abundance sensitivity	M+1/M <2 x 10^{-5}
Rinsing time	HNO_3-2 N + HF-0.01 N ~3 min
Typical blank contribution on ^{230}Th and ^{234}U	<1% and <1‰ respectively
